# Diffusion modeling of COVID-19 under lockdown

**DOI:** 10.1063/5.0044061

**Published:** 2021-04-12

**Authors:** Nicola Serra, Paola Di Carlo, Teresa Rea, Consolato M. Sergi

**Affiliations:** 1 Departments of Public Health, University Federico II of Naples, 80131 Naples, Italy; 2 Department of Health Promotion, Maternal-Childhood, Internal Medicine of Excellence “G. D'Alessandro,” PROMISE, University of Palermo, Palermo 90127, Italy; 3 Pathology Laboratories, Children's Hospital of Eastern Ontario, University of Ottawa, 401 Smyth Rd., Ottawa, Ontario K1H 8L1, Canada

## Abstract

Viral immune evasion by sequence variation is a significant barrier to severe acute respiratory syndrome coronavirus 2 (SARS-CoV-2) vaccine design and coronavirus disease-2019 diffusion under lockdown are unpredictable with subsequent waves. Our group has developed a computational model rooted in physics to address this challenge, aiming to predict the fitness landscape of SARS-CoV-2 diffusion using a variant of the bidimensional Ising model (2DIMV) connected seasonally. The 2DIMV works in a closed system composed of limited interaction subjects and conditioned by only temperature changes. Markov chain Monte Carlo method shows that an increase in temperature implicates reduced virus diffusion and increased mobility, leading to increased virus diffusion.

## INTRODUCTION

Coronaviruses are a large family of viruses known to cause diseases ranging from the common cold to more severe illnesses such as the Middle East respiratory syndrome (MERS) and severe acute respiratory syndrome (SARS). They are positive-stranded RNA viruses with a crown-like appearance under an electron microscope. The subfamily *Orthocoronavirinae* of the *Coronaviridae* family has been classified into four coronaviruses (CoV) genera: Alpha-, Beta-, Delta-, and Gamma-coronavirus. The betacoronavirus genus is further separated into five subgenres (including Sarbecovirus). Coronaviruses were identified in the mid-1960s and are known to infect humans and some animals. The primary target cells are the epithelial cells of the respiratory and gastrointestinal tracts. Seven coronaviruses are capable of infecting humans. There are more common human coronaviruses (HCoV) HCoV-OC43, HCoV-HKU1, HCoV-229E, and HCoV-NL63, causing the common cold and severe infections of the lower respiratory tract. Also, less common Betacoronaviruses such as SARS-CoV, MERS-CoV, and 2019-nCoV (currently called SARS-CoV-2) are known.^1–7^

SARS-CoV-2 is a new strain that has never been previously identified in humans and has been reported initially in Wuhan, China, in December 2019.^8–10^ Following the identification and labeling of the new coronavirus and the spread of the infection worldwide, The World Health Organization (WHO) declared a pandemic of COVID-19 (coronavirus disease-2019). Currently, more than 100 000 000 cases and more than 2 500 000 deaths are recorded worldwide. The death rate is 2.22%, while the WHO estimates that up to 650 000 people die of flu-related causes every year worldwide. The death rate for the flu is more difficult to calculate because the flu is not a reportable disease in most parts of the world.

Mathematical modeling is one of the most critical tools for analyzing infectious diseases' epidemiological characteristics and can provide some valuable insights into the disease dynamics. Various models have been used to study different aspects of the COVID-19 pandemic.^11–19^ Notably, recent studies have proposed mathematical models for predicting virus diffusion. Fortaleza CMCB *et al.* used geographic models of population mobility to check for patterns for the spread of SARS-CoV-2 infection in Brazil,^20^ while Siam *et al.* proposed an epidemiological model to predict the lockdown effect on COVID-19 diffusion in Bangladesh, showing that lockdown had a positive impact in reducing the virus diffusion. Still, it was disastrous for human welfare and national economies.^21^

Analogous results were obtained by Spelta *et al.*, using a simple model for the spreading disease represented by a Susceptible-Infected-Recovered (SIR) model.^22^ The positive lockdown impact on virus diffusion was confirmed by Lillery *et al.* in an Italian study, also.^23^

Also, Borro *et al.* proposed a computational fluid dynamic model.^24^ These authors evaluated the impact of ventilation and air conditioning systems on increasing or reducing the spreading of the infection in indoor environments.^24^

Giuliani *et al.* proposed an endemic-epidemic time-series mixed-effects generalized linear model for areal disease counts that have been implemented to understand and predict a spatiotemporal diffusion of COVID-19.^25^

Our study describes a mathematical diffusion model of COVID-19 under lockdown using a variant of a bidimensional (2D) Ising model (2DIMV). We describe the COVID-19 diffusion in a closed system composed of subjects with limited interactions due to lockdown conditions and considering only the temperature changes. For this scope, we used a variant of the 2D Ising model with external field *h* = 0 and the Markov chain Monte Carlo (MCMC) methods to simulate the systems considered.

## MATERIALS AND METHODS

### Ising model

Ising model is a straightforward and fundamental model in statistical mechanics, which can be used in several areas.^26–28^ It is often used to describe the phenomena of magnetization, liquid/gas coexistence, or in the image analysis as reported in recent papers.^29–32^

This model consisting of a lattice of “spin” variables *s*_i,_ which can only take the values +1(↑) o −1(↓). Every spin can interact with its nearest neighbors (2 in 1D) as well as with an external magnetic field *h*. The Hamiltonian is an operator that describes the full power of the Ising model,

$$H\left(\left\{s_{i}\right\}\right)=-J\sum_{\left\langle i,j\right\rangle }s_{i}s_{j}-h\sum_{i}s_{i},$$ (1)

where *J* is a constant specifying the strength of interaction, and the sum *⟨i,j⟩* is over nearest neighbors. The Ising model is usually studied in the canonical ensemble. In the canonical group, the probability of finding a particular spin configuration {*s*_i_} is as follows:

$$p\left(\left\{s_{i}\right\}\right)=\frac{1}{Z}e^{-\beta H\left(\left\{s_{i}\right\}\right)};\;\beta \equiv \frac{1}{k_{B}T},$$ (2)

where $Z=\sum_{\left\{s_{i}\right\}}e^{-\beta H\left(\left\{s_{i}\right\}\right)}$ is the partition function, and $e^{-\beta H\left(\left\{s_{i}\right\}\right)}$ represents the Boltzmann factor that induce to prefer spin configurations with lower energies in an Ising model. Particularly, it describes the effect of *h* and *J* on the behavior of the spins. With regard to *h,* when *h* > 0, *s_i_* = +1 is favored, while when *h* < 0, *s_i_* = -1 is favored. This aspect means that the spins want to align with the direction of *h*. With regard to *J*, when *J* > 0, neighboring spins prefer to be parallel (i.e., *s_i_* = +1 and *s_i+_*_1_ = +1 or *s_i_* = -1 and *s_i+_*_1_ = –1), while when *J* < 0, neighboring spins prefer to be anti-parallel (i.e., *s_i_* = +1 and *s_i+_*_1_ = −1 or *s_i_* = –1 and *s_i+_*_1_ = +1).

Also, in the 2D Ising model at low enough temperature, all spins will cooperate and spontaneously align themselves (e.g., most spins become +1) even in the absence of the external field (*h* = 0).^33–36^ This phenomenon is called “spontaneous magnetization.” At high enough temperatures, the spontaneous magnetization is destroyed by thermal fluctuation. Hence the 2D Ising model has a critical temperature *Tc*, below which a spontaneous magnetization occurs and above which it does not occur. In other words, there is a transition phase at *Tc*, from ordered (*T* < *Tc*) to disordered phase (*T > Tc*).

### Lockdown conditions

The confinement measures, containment measures, and blocking actions, also indicated with the lockdown anglicism, constitute an emergency protocol that imposes restrictions on people's free movement for various reasons, ranging from health related to public safety issues. Recently, many countries have used this protocol to limit COVID-19 diffusion.

### 2D Ising model variant for modeling of COVID-19 diffusion under lockdown conditions

To describe the COVID-19 diffusion in a city with lockdown conditions, we used a 2D Ising model variant (2DIMV-COVID-19), where the fundamental parameters, like *temperature (T)*, *magnetization* (*M*), and *energy (E)*, were reinterpreted in *temperature*, *diffusion,* and *mobility,* respectively. In particular, about the mobility, we considered the spins with more energy as spins with more capacity of movement or vibration in comparison to others, regarding a lattice structure, and therefore associable to subjects with more but limited mobility, similar to the lockdown conditions.

About the magnetization, we considered the “spin” variables *s*_i_ with a value equal to −1 as a subject negative to COVID-19, i.e., a subject in health status, while a “spin” variables *s*_i_ with a value equal to +1 as a subject positive/infected with COVID-19. Mainly the temperature was considered variable into range 0 °C–42 °C. In other words, we considered the seasonality, i.e., winter-spring-summer, because it is connected to virus diffusion, such as other similar infections. We introduced a statistical parameter λ to characterize the seasonality and virus diffusion. It considers the seasonality and virus diffusion connected to temperature change. We obtained it considering the distribution of similar viruses associated with respiratory deficiency, such as influenza, infective diseases of the upper and lower respiratory tract, etc.^37–39^ This parameter described in (3) is a probability distribution represented by the Gaussian distribution and defined by

$$\lambda =ae^{-\frac{1}{2}{\left(\frac{\mu -x}{\sigma }\right)}^{2}},\;a=1;\;\mu =10;\;\sigma =10.$$ (3)

**FIG. 1.**
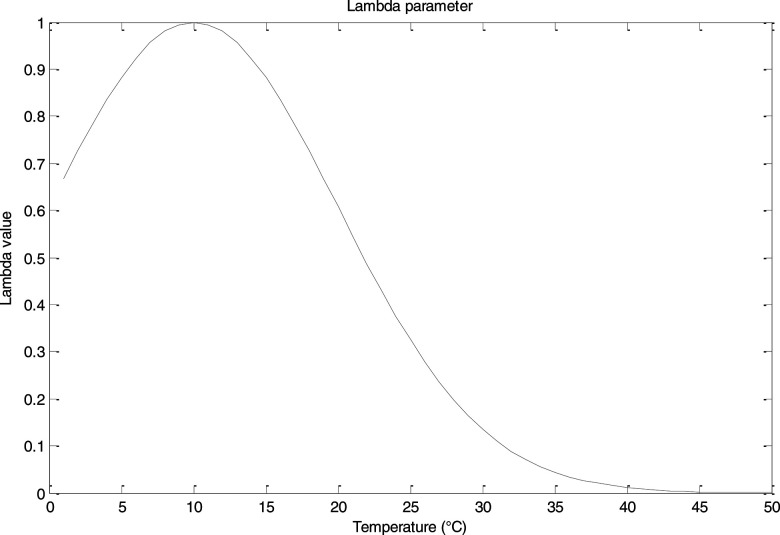
Lambda (λ) parameter distribution.

We show in Fig. 1 the λ parameter distribution.

It is possible to observe that the lambda parameter increases from 0 °C to about 10 °C and decreases to 50 °C, but we consider in our model 42 °C as the maximum value.

### Simulating 2DIMV-COV19 with Markov chain Monte Carlo method

We considered a 2D Ising model variant defined over a square lattice of 400 “spins” variables is in this step. The Hamiltonian is again equal to Eq. (1). *J* describes the strength of interaction, while *h* is the external magnetic field. The sum ⟨*s_i_*,*s_i_*⟩ is the nearest-neighbor pairs, considering no or impaired mobility connected to lockdown status. Mainly, periodic boundary conditions have been applied. The spins on one edge of the lattice are neighbors of the corresponding spins on the other side. This aspect ensures that all spins have the same number of neighbors and local geometry and that there are no distinctive edge spins with different properties from the others. All the spins are equivalent, and the system is entirely translationally invariant. The MCMC algorithm is a simple and widely used approach to generate the canonical ensemble. It is convenient to implement the 2DIMV. The initial configuration is characterized by the strength of interaction *J* = 1, the Boltzmann constant *k* = 1, and the external field *h* equal to zero in the Hamiltonian distance. It indicates, in the initial configuration, that all subjects are healthy (all spins had a value equal to −1). In the event *T* increases, the thermal energy *kTλ* available to flip the spins is infinitely more massive than the energy due to the spin-spin interaction *J*, so the spins are oriented randomly up or down in an uncorrelated fashion.

The simulations are often performed consecutively in a range of different *T* values, particularly for all values of *T*. The system's initial state is represented by an ensemble of spins with instantaneous magnetization equal to –1, i.e., associates with sane subjects. The first step in the simulation is to generate a new state that should differ from the present one by the flip of one spin (an algorithm that does this is said to have single-spin-flip dynamics). Every such state should be exactly as likely as every other to be generated. This aspect is accomplished by picking, at random, a single spin *p* from the lattice to be flipped. The difference in energy between the new state and the old is then calculated. The only term in the first term of the Hamiltonian that change involves the flipped spin.

The others remain unchanged and so cancel out when the difference *Ev* – *Eu* is taken. The change in energy between the two states is as follows:

$$E_{v}-E_{u}=-J\sum_{\left\langle i,j\right\rangle }s_{i}^{v}s_{j}^{v}+J\sum_{\left\langle i,j\right\rangle }s_{i}^{u}s_{j}^{u}=-J\sum s_{i}^{u}\left(s_{p}^{v}-s_{p}^{u}\right).$$ (4)

In the second line, the sum is over only those spins *i* of the nearest neighbors of the flipped spin *p,* and all of the spins do not themselves flip, so that: $s_{i}^{v}=s_{i}^{u}$. Particularly,

if $s_{p}^{u}=+1$ then after spin *p* has been flipped $s_{p}^{v}=-1$, so that: $s_{p}^{v}-s_{p}^{u}=-2$;

if $s_{p}^{u}=-1$ then after spin *p* has been flipped $s_{p}^{v}=-1$, so that: $s_{p}^{v}-s_{p}^{u}=+2$.

Thus, we have $s_{p}^{v}-s_{p}^{u}=-2s_{p}^{v}$, and

$$\Delta E=E_{v}-E_{u}=2J\sum s_{i}^{u}s_{p}^{u}=2Js_{p}^{u}\sum s_{i}^{u}.$$ (5)

This aspect involves summing over four terms in a square lattice case, where the nearest neighbors are the squares above, below, left, and right. If a new state is selected, which has energy lower than or equal to the current, then the transition to that state should always be accepted. If it has higher energy, then it may be obtained according to the probability $e^{-\frac{\Delta E}{kT}}$, where *k* is the Boltzmann constant. The temperature is measured in energy units, so that *k* = 1. This is done by choosing a random number 0 ≤ *n* < 1. If the random number is less than the probability, i.e., *n <* $e^{-\frac{\Delta E}{kT}}$, then the spin is flipped; otherwise, the spin remains unchanged.^6^ This selection method uses the Metropolis algorithm introduced by Metropolis *et al.* in the simulation of gas particles, where each particle was considered a solid sphere.^40–43^ In our 2DIMV-COVID-19, the prior probability described changes in $e^{-\frac{\Delta E}{kT\lambda }}$, where *λ* represents the statistical parameter introduced in (3).

The simulation phase begins with the same initial configuration for each temperature value, i.e., all subjects are healthy. All sites are blank or with instantaneous magnetization equal to −1 with the MCMC method. For each temperature value, a group of 12 000 spin configurations is generated until T = 42 °C, with a total of 1 020 000 spin configurations generated for this study. We observed that the temperature is the independent parameter, while the magnetization, and internal energy, are dependent parameters, i.e., they vary in dependence on the temperature only.

Our simulation begins by considering the initial temperature equal to zero (*T*= 0). Considering a lattice where for every site, the spin has an instantaneous magnetization value equal to −1, mainly this condition was represented in the simulation with a lattice site in black.

Increasing the temperature, some spins change their state. This event is crucial for the interpretation. Initially, every spin, with an instantaneous magnetization value equal to –1, could be associated with a healthy subject (black site). In contrast, a change status, i.e., a magnetized spin with reverse orientation (blank spot) or value equal to 1 of instantaneous magnetization, could be interpreted with a subject positive/infected by COVID-19.

## RESULTS

**FIG. 2.**
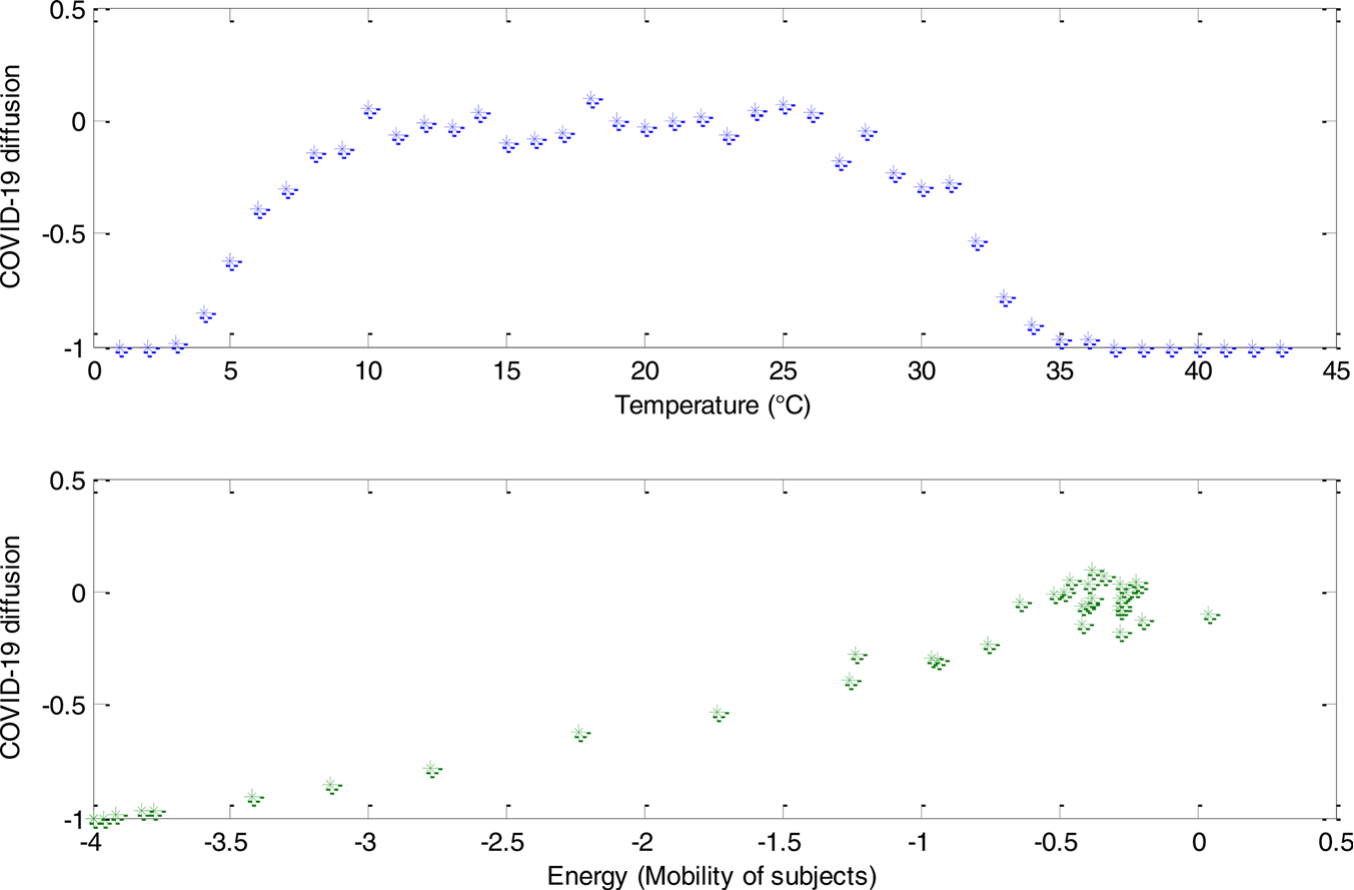
COVID-19 diffusion, in relationship to temperature [(a), *upper graphic*] and mobility [(b*)*, *lower graphic*].

In Figs. 2(a) and 2(b), we reported mean magnetization simulations depending on both temperature and mean internal energy. The mean magnetization was obtained considering the mean of all instantaneous magnetizations of the 20 ensemble spin configurations obtained for each temperature value. Analogous for internal energy.

**FIG. 3.**
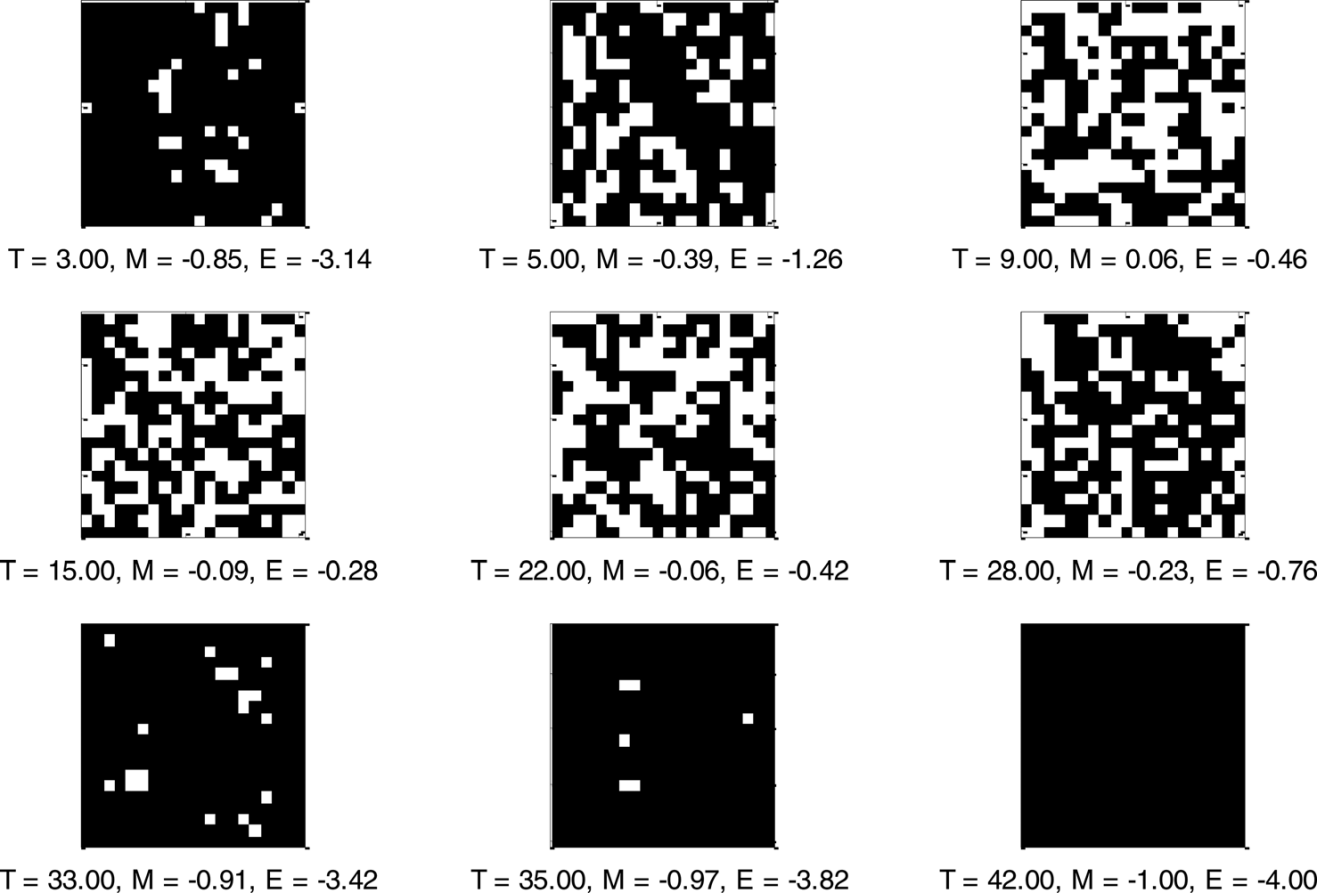
MCMC simulations of 2D Ising model variant at a different temperature, considering a square lattice of 400 “spins.” In the figure were reported the temperature (T), mean magnetization (M), and mean internal energy (E).

From Fig. 2(a) (upper graphic), we observed that considering a closed system, i.e., a city isolates and under lockdown conditions, the virus diffusion increases rapidly between about 2.5 and 10 °C. Subsequently, there is an equilibrium between the number of infected and no infected, with minor fluctuations between 10 and 25 °C. From 26 °C to 37 °C, there is a gradual reduction of infected or positive to COVID-19. Finally, after 37 °C, the positive/infected subjects lean to zero. Figure 2(b) (lower graphic) shows the relationship between COVID-19 diffusion and mobility. It is possible to observe that an increase in mobility under lockdown conditions implicates increased virus diffusion. In other words, the absent or reduced mobility contributes to a decrease in contagiousness. Significantly, maximum internal energy, i.e., the whole possibility of movement in lockdown conditions, is associated with the maximum number of positive/infected subjects (Fig. 3). In Fig. 3, we showed some simulated system configurations at different temperatures.

## DISCUSSION

Mathematical models represent a powerful tool in predicting and describing events and are recently used in predicting COVID-19 diffusion. In the fight against COVID-19, it is crucial to find out how to quickly overcome this virus and save as many human lives as possible. For this purpose, researchers in every field try to contribute to stop or contain the pandemic. In this direction, strong collaborations among researchers with different skills are consolidated. Numerous mathematical models are being produced to forecast the future of COVID-19 epidemics worldwide, as described in a recent overview, particularly if the virus becomes seasonal.^44^

In this paper, we propose the forecast of COVID-19 diffusion considering a close system and lockdown condition to evaluate the impact of this restriction and the temperature change on the diffusion. For this scope, a 2D Ising model variant was considered. In this case, the 2D Ising model's use was adequate in describing the virus diffusion in lockdown status in a closed system considering the seasonally and consequently the temperature only as an independent variable. In fact, we hypnotized a closed system with reduced mobility (for example, a city where the lockdown is active) correspondent at a two-dimensional lattice of spins, where the spins (subjects) can only take two values −1= healthy and +1= positive/infected. Also, the lattice is a rigid structure. Therefore, every spin can interact with its nearest neighbors. In this way, the virus diffusion can be interpreted analogously to the phase transition in the Ising model when spins change state under the magnetization effect.

MCMC algorithm was used to simulate the probabilistic canonical ensemble considering different *T* values, starting from the same initial configuration represented by all subjects sane.

In a recent study, the Ising model was used by Padhi *et al.*^45^ to compare their SIRD model, used to analyze the extent to which this multi-phased lockdown has been active in flattening the diffusion curve and lower the threat. Notably, the authors emphasize how the Ising model could be used combined with a quantum computational approach, considering time as an independent variable.

Our results show that with a temperature less than about 2.5 °C, the virus diffusion is null. This aspect could be not only to reduced mobility of the subjects lead to lockdown status, but it could be due to low propensity of movement at very low temperatures. Consequently, there is minimal virus diffusion. With a *T* > *T*_c_ (about 2.5 °C), we observed that by starting all subjects as healthy, the virus diffusion rapidly increased between about 2.5–10 °C. In contrast, between about 10–25 °C, the diffusion is at maximum and similar to the range. After 25 °C, there is a gradual decrease in virus diffusion until 37 °C. Finally, after 37 °C the virus diffusion tends to zero, showing the seasonal incidence on COVID-19 diffusion. We underline that these temperature values should be considered reference values and orientation values that guide the impact of seasonality on COVID-19 diffusion.

Additional considerations are connected to the mobility of the subjects on virus diffusion. Significantly an increase in mobility is connected to the possibility of interactions among subjects. Consequently, our results show that the COVID-19 diffusion is maximum in relation to full mobility admitted in lockdown status. Our results are comparable to some modeling data.^44^ Still, in our modeling of COVID-19 diffusion, we considered the lockdown status, i.e., the reduced mobility of subjects and the seasonally, not considered in other studies.

In conclusion, the results obtained in this study may be highly relevant now that several countries are contemplating a lockdown at the national level due to the emergency of virus variants and decide the best time to activate the lockdown.

## Data Availability

The data that support the findings of this study are available from the corresponding author upon reasonable request.

## References

[1] M. Ciotti , S. Angeletti , M. Minieri , M. Giovannetti , D. Benvenuto , S. Pascarella , C. Sagnelli , M. Bianchi , S. Bernardini , and M. Ciccozzi , “ COVID-19 outbreak: An overview,” Chemotherapy 64, 215–219 (2019). 10.1159/000507423 32259829 PMC7179549

[2] S. Ludwig and A. Zarbock , “ Coronaviruses and SARS-CoV-2: A brief overview,” Anesth. Analg. 131(1), 93–96 (2020). 32243297 10.1213/ANE.0000000000004845 PMC7173023

[3] Z. W. Ye , S. Yuan , K. S. Yuen , S. Y. Fung , C. P. Chan , and D. Y. Jin , “ Zoonotic origins of human coronaviruses,” Int. J. Biol. Sci. 16, 1686–1697 (2020). 10.7150/ijbs.45472 32226286 PMC7098031

[4] M. Xie and Q. Chen , “ Insight into 2019 novel coronavirus—An updated intrim review and lessons from SARS-CoV and MERS-CoV,” Int. J. Infect. Dis. 94, 119–124 (2020). 32247050 10.1016/j.ijid.2020.03.071 PMC7118633

[5] M. Zhou , X. Zhang , and J. Qu , “ Coronavirus disease 2019 (COVID-19): A clinical update,” Front. Med. 14(2), 126–135 (2020). 32240462 10.1007/s11684-020-0767-8 PMC7115348

[6] H. Li , Y. Zhou , M. Zhang , H. Wang , Q. Zhao , and J. Liu , “ Updated approaches against SARS-CoV-2,” Antimicrob. Agents Chemother. 64(6), e00483-20 (2020). 32205349 10.1128/AAC.00483-20 PMC7269512

[7] Z. J. Cheng and J. Shan , “ 2019 Novel coronavirus: Where we are and what we know,” Infection 48, 155–163 (2020). 10.1007/s15010-020-01401-y 32072569 PMC7095345

[8] S. E. Park , “ Epidemiology, virology, and clinical features of severe acute respiratory syndrome -coronavirus-2 (SARS-CoV-2; coronavirus disease-19),” Clin. Exp. Pediatr. 63, 119 (2020). 10.3345/cep.2020.00493 32252141 PMC7170784

[9] C. Wang , Z. Liu , Z. Chen , X. Huang , M. Xu , T. He , and Z. Zhang , “ The establishment of reference sequence for SARS-CoV-2 and variation analysis,” J. Med. Virol. 92, 667 (2020). 10.1002/jmv.25762 32167180 PMC7228400

[10] X. Jiang , S. Rayner , and M. H. Luo , “ Does SARS-CoV-2 has a longer incubation period than SARS and MERS?,” J. Med. Virol. 92, 476–478 (2020). 10.1002/jmv.25708 32056235 PMC7166592

[11] S. Sanche , Y. T. Lin , C. Xu , E. Romero-Severson , N. Hengartner , and R. Ke , “ High contagiousness and rapid spread of severe acute respiratory syndrome coronavirus 2,” Emerg. Infect. Dis. 26(7), 1470–1477 (2020). 32255761 10.3201/eid2607.200282 PMC7323562

[12] X. M. Rong , L. Yang , H. D. Chu , and M. Fan , “ Effect of delay in diagnosis on transmission of COVID-19,” Math. Biosci. Eng. 17, 2725–2740 (2020). 10.3934/mbe.2020149 32233563

[13] F. Rahimi and A. Talebi Bezmin Abadi , “ Practical strategies against the novel coronavirus and COVID-19-the imminent global threat,” Arch. Med. Res. 51(3), 280–281 (2020). 32229157 10.1016/j.arcmed.2020.03.005 PMC7270650

[14] K. Prem , Y. Liu , T. W. Russell , A. J. Kucharski , R. M. Eggo , N. Davies , C.-W. G. Centre for the Mathematical Modelling of Infectious Diseases, M. Jit , and P. Klepac , *The Effect of Control Strategies to Reduce Social Mixing on Outcomes of the COVID-19 Epidemic in Wuhan, China: A Modelling Study* ( Lancet Public Health, 2020). 10.1016/S2468-2667(20)30073-6 PMC7158905 32220655

[15] B. Robson , “ Computers and viral diseases. Preliminary bioinformatics studies on the design of a synthetic vaccine and a preventative peptidomimetic antagonist against the SARS-CoV-2 (2019-nCoV, COVID-19) coronavirus,” Comput. Biol. Med. 119, 103670 (2020). 10.1016/j.compbiomed.2020.103670 32209231 PMC7094376

[16] A. J. Kucharski , T. W. Russell , C. Diamond , Y. Liu , J. Edmunds , S. Funk , R. M. Eggo , C.-W. G. Centre for Mathematical Modelling of Infectious Diseases, “ Early dynamics of transmission and control of COVID-19: A mathematical modelling study,” Lancet Infect. Dis. 20(5), 553–558 (2020). 32171059 10.1016/S1473-3099(20)30144-4 PMC7158569

[17] J. Hellewell , S. Abbott , A. Gimma , N. I. Bosse , C. I. Jarvis , T. W. Russell , J. D. Munday , A. J. Kucharski , W. J. Edmunds , C.-W. G. Centre for the Mathematical Modelling of Infectious Diseases , S. Funk , and R. M. Eggo , “ Feasibility of controlling COVID-19 outbreaks by isolation of cases and contacts,” Lancet Glob. Health 8, e488–e496 (2020). 10.1016/S2214-109X(20)30074-7 32119825 PMC7097845

[18] T. M. Chen , J. Rui , Q. P. Wang , Z. Y. Zhao , J. A. Cui , and L. Yin , “ A mathematical model for simulating the phase-based transmissibility of a novel coronavirus,” Infect. Dis. Poverty 9, 24 (2020). 10.1186/s40249-020-00640-3 32111262 PMC7047374

[19] K. Gostic , A. C. Gomez , R. O. Mummah , A. J. Kucharski , and J. O. Lloyd-Smith , “ Estimated effectiveness of symptom and risk screening to prevent the spread of COVID-19,” eLife 9, e55570 (2020). 10.7554/eLife.55570 32091395 PMC7060038

[20] C. Fortaleza , R. B. Guimaraes , R. C. Catao , C. P. Ferreira , G. Berg de Almeida , T. Nogueira Vilches , and E. Pugliesi , “ The use of health geography modeling to understand early dispersion of COVID-19 in Sao Paulo, Brazil,” PLoS One 16, e0245051 (2021). 10.1371/journal.pone.0245051 33411768 PMC7790416

[21] Z. S. Siam , M. Arifuzzaman , M. S. Ahmed , F. A. Khan , M. H. Rashid , and M. S. Islam , “ Dynamics of COVID-19 transmission in Dhaka and Chittagong: Two business hubs of Bangladesh,” Clin. Epidemiol. Glob. Health 10, 100684 (2021). 10.1016/j.cegh.2020.100684 33392419 PMC7759449

[22] A. Spelta , A. Flori , F. Pierri , G. Bonaccorsi , and F. Pammolli , “ After the lockdown: Simulating mobility, public health and economic recovery scenarios,” Sci. Rep. 10, 16950 (2020). 10.1038/s41598-020-73949-6 33046737 PMC7550600

[23] D. Lilleri , F. Zavaglio , E. Gabanti , G. Gerna , and E. Arbustini , “ Analysis of the SARS-CoV-2 epidemic in Italy: The role of local and interventional factors in the control of the epidemic,” PLoS One 15, e0242305 (2020). 10.1371/journal.pone.0242305 33180880 PMC7660511

[24] L. Borro , L. Mazzei , M. Raponi , P. Piscitelli , A. Miani , and A. Secinaro , “ The role of air conditioning in the diffusion of Sars-CoV-2 in indoor environments: A first computational fluid dynamic model, based on investigations performed at the Vatican State Children's hospital,” Environ. Res. 193, 110343 (2021). 10.1016/j.envres.2020.110343 33068577 PMC7557177

[25] D. Giuliani , M. M. Dickson , G. Espa , and F. Santi , “ Modelling and predicting the spatio-temporal spread of cOVID-19 in Italy,” BMC Infect. Dis. 20, 700 (2020). 10.1186/s12879-020-05415-7 32967639 PMC7509829

[26] T. Ezaki , T. Watanabe , M. Ohzeki , and N. Masuda , “ Energy landscape analysis of neuroimaging data,” Philos. Trans. A: Math. Phys. Eng. Sci. 375, 20160287 (2017). 10.1098/rsta.2016.0287 28507232 PMC5434078

[27] C. Ma , H. H. Zhang , and X. Wang , “ Machine learning for big data analytics in plants,” Trends Plant Sci. 19, 798–808 (2014). 10.1016/j.tplants.2014.08.004 25223304

[28] M. Hogyoku , “ Criticality found in a model for orientational ordering of protein arrays,” Adv. Biophys. 34, 55–68 (1997). 10.1016/S0065-227X(97)89631-1 9204126

[29] M. Kawamura , K. Hayashi , T. Uezu , and M. Okada , “ Statistical mechanical evaluation of a spread-spectrum watermarking model with image restoration,” Phys. Rev. 99, 062132 (2019). 10.1103/PhysRevE.99.062132 31330627

[30] E. J. Davis , A. Periwal , E. S. Cooper , G. Bentsen , S. J. Evered , K. Van Kirk , and M. H. Schleier-Smith , “ Protecting spin coherence in a tunable heisenberg model,” Phys. Rev. Lett. 125, 060402 (2020). 10.1103/PhysRevLett.125.060402 32845652

[31] H. Arian Zad and N. Ananikian , “ Phase transitions and magnetization of the mixed-spin Ising-Heisenberg double sawtooth frustrated ladder,” J. Phys.: Condens. Matter 30, 165403 (2018). 10.1088/1361-648X/aab644 29589591

[32] A. A. B. Pessa and H. V. Ribeiro , “ Mapping images into ordinal networks,” Phys. Rev. E 102, 052312 (2020). 10.1103/PhysRevE.102.052312 33327134

[33] W. Zhong , G. T. Barkema , and D. Panja , “ Super slowing down in the bond-diluted Ising model,” Phys. Rev. E 102, 022132 (2020). 10.1103/PhysRevE.102.022132 32942400

[34] F. C. Thewes and H. C. M. Fernandes , “ Phase transitions in hard-core lattice gases on the honeycomb lattice,” Phys. Rev. E 101, 062138 (2020). 10.1103/PhysRevE.101.062138 32688552

[35] A. Pimachev , R. D. Nielsen , and Y. Dahnovsky , “ High-temperature 2D ferromagnetism in conjugated microporous porphyrin-type polymers,” Phys. Chem. Chem. Phys. 22, 14480–14488 (2020). 10.1039/D0CP02312D 32568338

[36] M. P. Zaletel and F. Pollmann , “ Isometric tensor network states in two dimensions,” Phys. Rev. Lett. 124, 037201 (2020). 10.1103/PhysRevLett.124.037201 32031848

[37] J. Yan , S. Guha , P. Hariharan , and M. Myers , “ Modeling the effectiveness of respiratory protective devices in reducing influenza outbreak,” Risk Anal. 39, 647–661 (2019). 10.1111/risa.13181 30229968

[38] R. Rosselli , M. Martini , G. Fluad Effect Working , N. L. Bragazzi , and A. Watad , “ The public health impact of the so-called "fluad effect" on the 2014/2015 influenza vaccination campaign in Italy: Ethical implications for health-care workers and health communication practitioners,” Adv. Exp. Med. Biol. 973, 125–134 (2017). 10.1007/5584_2017_39 28452003

[39] T. Jefferson , C. Del Mar , L. Dooley , E. Ferroni , L. A. Al-Ansary , G. A. Bawazeer , M. L. van Driel , R. Foxlee , and A. Rivetti , “ Physical interventions to interrupt or reduce the spread of respiratory viruses: Systematic review,” BMJ 339, b3675 (2009). 10.1136/bmj.b3675 19773323 PMC2749164

[40] F. Faizi , G. Deligiannidis , and E. Rosta , “ Efficient irreversible Monte Carlo samplers,” J. Chem. Theory Comput. 16, 2124–2138 (2020). 10.1021/acs.jctc.9b01135 32097548

[41] T. Cary , R. R. P. Singh , and R. T. Scalettar , “ Tricriticality in crossed Ising chains,” Phys. Rev. E 96, 042108 (2017). 10.1103/PhysRevE.96.042108 29347573

[42] A. Jedrzejewski , A. Chmiel , and K. Sznajd-Weron , “ Kinetic Ising models with various single-spin-flip dynamics on quenched and annealed random regular graphs,” Phys. Rev. E 96, 012132 (2017). 10.1103/PhysRevE.96.012132 29347245

[43] N. Stanica , F. Cimpoesu , C. Radu , V. Chihaia , and S. H. Suh , “ Monte Carlo simulations of the magnetic behavior, ordering temperature and magnetocaloric effects in 1D, 2D and 3D ferrimagnetic systems,” J. Nanosci. Nanotechnol. 15, 263–268 (2015). 10.1166/jnn.2015.8416 26328343

[44] D. Adam , “ Special report: The simulations driving the world's response to COVID-19,” Nature 580, 316 (2020). 10.1038/d41586-020-01003-6 32242115

[45] A. Padhi , S. Pradhan , P. P. Sahoo , K. Suresh , B. K. Behera , and P. K. Panigrahi , “ Studying the effect of lockdown using epidemiological modelling of COVID-19 and a quantum computational approach using the Ising spin interaction,” Sci. Rep. 10, 21741 (2020). 10.1038/s41598-020-78652-0 33303815 PMC7729942

